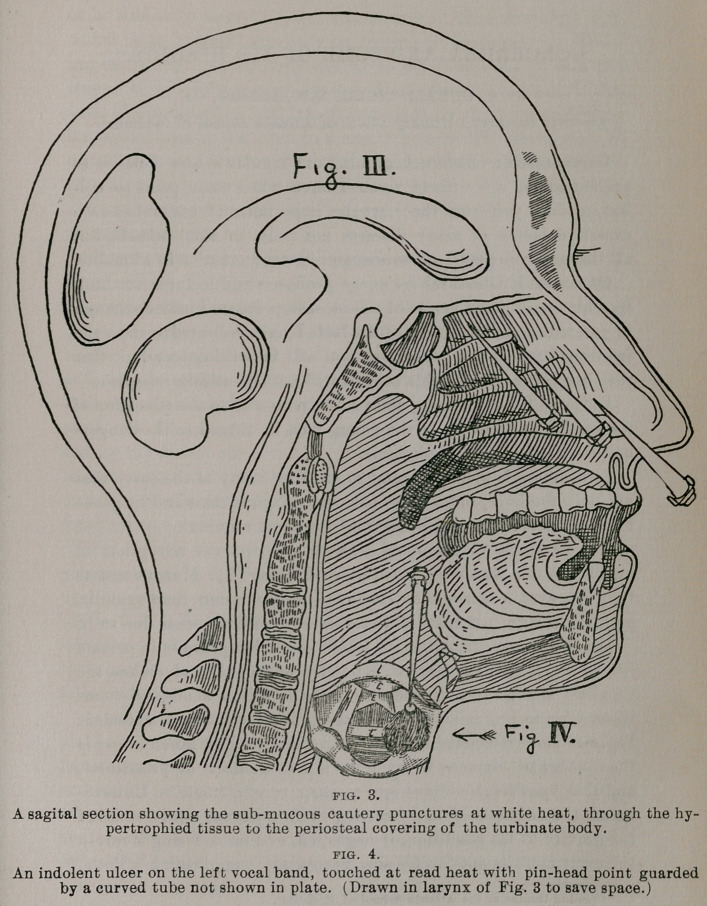# Some of the Uses and Abuses of the Galvano-Cautery

**Published:** 1906-02

**Authors:** Arthur G. Hobbs

**Affiliations:** Atanta, Ga.


ATLANTA
### Journal-Record of Medicine

Successor to Atlanta Medical and Surgical Journal, Established 1855, and Southern Medical Record, Established 1870.

OWNED BY THE ATLANTA MEDICAL JOURNAL CO.

Published Monthly.

VOL. VII. FEBRUARY, 1906. No. 11.

BERNARD WOLFF, M.D., EDITOR, Nos. 319–20 Prudential.

M. B. HUTCHINS, M.D., BUSINESS MANAGER, 1007–1008 Century Bldg.

E. G. BALLENGER, M.D., ASSOCIATE EDITOR AND ASSISTANT MANAGER.

J. N. LECONTE, M.D., FOREIGN CORRESPONDENT.

### ORIGINAL COMMUNICATIONS.

### SOME OF THE USES AND ABUSES OF THE GALVANO-CAUTERY.

WITH SPECIAL REFERENCE TO ITS SYSTEMIC RESULTS WHEN APPLIED LOCALLY TO THE UPPER RESPIRATORY TRACT; TO ITS ADAPTATION TO SINUS COMPLICATIONS; AND ALSO TO ITS LOCAL USES IN SOME EYE AND EAR CASES.

By ARTHUR G. HOBBS, M. D., ATLANTA, GA.

In 1893 I wrote a paper on the hypodermic (more properly the hypomucous) application of the galvano-cautery with the object of buttoning down an hypertrophy, or an hyperplasia over the enlarged turbinates.

The paper was tentative in character, as others than myself were, at that time, trying to arrive at some means of producing a periosteal cicatrix that would draw down these thickened tissues, and thus add to the nasal lumen and at the same time leave the mucous membrane intact.

In 1894 I wrote another paper on the use of this cautery in pterigia and, incidentally, on its use in the shape of a small blunt point, in corneal ulcers.

In 1896 again I published a short paper on the cautery's possibilities in the form of a loop for excising tonsils, particularly in patients with a hemorrhagic history; and also with its point blunted and curved for going to the bottom of tonsillar crypts, containing cheesy and malodorous exudations. Since writing this last paper I have used the loop for extracting postnasal polypi in bleeders, by substituting a small platinum wire in place of the ordinary piano wire of the thumb ecraseurs. It is also well to resort to this method in any case of persistently recurring polypus, as it sears the pedicle, and lessens the chances of regrowth.

About this date, in a paper on septum operations, I especially mentioned the use of the blunt point as one of the best means of stopping a syphilitic invasion of the cartilaginous or bony parts of the septum (the incentive was a recent resort to it for an exposed spinous process, necrosed at the bottom of a pharyngeal ulcer).

Again, after this, in a paper on middle ear diseases, I described its advantages over caustics when applied in the form of a needle point, to protruding granulations, or to old and persisting polyp roots.

I now think that the point may, advantageously and without danger, be applied to almost any persistent ulcerative process, particularly when the necrosed tissue has its base at the periosteum.

Any agent of such potency as the galvano-cautery is quite as capable of doing harm in ignorant and untried hands as it can be made to do good when properly used. But this is an old axiom especially applied because it is even more true here than elsewhere.

I can only allude to some of the abuses of this agent: It certainly would be an abuse to use it at a cherry or red heat, when the white is the best. The cherry or red heat is to destroy necrosed tissue and to stimulate the contiguous normal tissues, and the white to produce a subcicatrix to cut off the nutrition, and to result in reduction by atrophy and cicatrix. It would be an abuse of this agent to make deep parallel or crucial incisions, and still worse to apply the flat blade to protruding turbinals. Either would produce a surface scar and pervert the natural functions of the mucous membrane. I might have said it does produce this result, as I often see, at my first examination, that this has been done. Again it would be an abuse to burn the pillars of the fauces, or the root of the tongue when excising a tonsil, or to scorch the nose or the ear when withdrawing the instrument from the deeper parts of these canals. Yet again it would be inexcusable to pierce the anterior chamber of the eye when applying the cherry heat to a corneal ulcer, nor should the sclera ever be burned when cutting through the neck of a pterigium.

To save the mucous membrane should be the first thought of him who invades the nasopharyngeal region with a cautery. It is easy to see why: The superficial area of the mucous membrane of the nasopharynx is measured by some at twenty-eight square inches, and by others at thirty, but Onodi, one of the best authorities, gives it at thirty-two square inches from the vocal chords outwards. This author further states that this surface will secrete two pints of fluid in twenty-four hours in a cold dry atmosphere, and probably only two to four ounces when it is warm and humid. Again, when it is cold and dry, this membrane appears congested and swollen, and on the contrary, when the atmosphere is warm and moist the whole tract looks blanched and the nasal lumen is greater. In short the automatic adaptation of this upper respiratory membrane is, all things considered, not surpassed by any other of the organs of involition. It warms and moistens the inspired air only when needed, and in proper proportion. Hence this membrane should be preserved, and it can be done after the proper use of the white point.

The mixed columnar and columnar-ciliated variety of mucous membrane, which we find over the turbinals and lining of the upper pharynx is more easily destroyed than the squamous variety that covers the lower pharynx, pillars, and tonsils. The former was not intended for rough usage while fulfilling its own distinctive functions, but the deglutition part of the pharynx membrane was made to resist the rough contact of the bolus.

The dry and parched throat of the mouth-breather is one of the first evidences of any interference with this automatism. Nature only intended the mouth to be used as an auxiliary channel in lieu of the temporary closure of the nasal or natural way. The mucous membrane surface, over which the inspired air passes by the auxiliary route, is so small as compared to that of nature's way, that the double function of warming and moistening the air is imperfectly performed.

The dry and parched throat is only a mild though significant suggestion of the more serious results that always follow any prolonged perversion of these functions. The continued passing of the unprepared air over the bronchial mucoci is not local only in its effects but further than this: Oxidation, secretion, excretion, nutrition, and in fact all the metabolic processes may be interrupted, as is so often evidenced by the pallor, the pinched face, and the stunted growth in mouth-breathing children; and adults often show no less decided systemic effects from long-continued nasal stenoses, even where all probabilities of nerve reflexes are excluded. The frequent restorations to normal healthy growth in children, and to vigor and increased weight in adults after restoring these normal functions of the upper respiratory tract, never cease to be surprising, although they are only logical sequences. Remove the local—the physical—cause and the systemic sequelæ are cured. Turbinal hypertrophies are primary and local; most frequently found in full-blooded—sthenic—subjects; rarely in the discrasias, certainly not in tuberculosis or syphilis, nor even in struma where the tonsils are often enlarged as glands; neither do any of the zymotic or any other of the systemic diseases cause turbinal hypertrophies. Hence this local condition should be corrected, not only for the comfort of good breathing, but for the still better systemic results. Certainly then this condition is regarded too lightly by many diagnosticians as an etiological factor in many systemic perversions, as well in adults as in growing children.

Again, is it not possible that these normally profuse secretions of the upper respiratory tract may effect some antiseptic change in the inspired air, in addition to mechanically filtering, and to warming and moistening it? And now in granting these far-reaching effects of nasal stenoses, what is the conclusion? It is, I think, that the proper use of the galvano-cautery is the best direct means for their correction. It goes to the root of the trouble, and its results are permanent. Hypertrophies can never recur at the site of a well-applied white point. The dimple permanently remains. Since the advent of the suprarenal extracts, it is easier to reach the superior turbinate, with the white point in treating the accessory sinus complications.

When applied to an indolent corneal ulcer, the point of the cautery should be blunted to the size and shape of the head of a pin, and the touch made quickly at cherry heat. The head of the patient should be held firmly, with the wrist of the operating hand resting on the forehead or cheek of the patient. The result is quite satisfactory in that this method leaves less opacity than probably any other of the treatments for such corneal ulcers. (I see two physicians before me on whom I have made this little operation, and neither now has even a leucoma as a result.)

It is the quickest and least troublesome method of operating on a pterigium with a narrow neck under which a grooved director can be slipped to protect the sclera as the neck is severed with a small cherry blade. In applying the cherry heat to persistent ulcers about the vocal chords I improvise a protecting tube for the shank bent to the proper curve for each case.

Its use in aural practice is not confined to its application to protrusions through the broken membrana tympani, since it may be resorted to with an ultimate good to the ear by reducing any hypertrophy or hyperplasia around the nasal end of the Eustachian tube, even though the swelling may not be sufficient to cause nasal stenosis. It is well-known how difficult it is, and how it is sometimes impossible to reach the Eustachian tube until the lip-like thickenings around its nasal end are reduced.

Again, the advantages of this hypo-mucus method of using the white point are not confined to the soft parts that cover the turbinates, since the necessity of sawing off these bony enlargements can often be obviated by pushing the point through the periosteal covering into the soft and friable bone, when the resulting atrophy will be no less marked than that of the softer tissues. Hence the more serious and objectionable sawing operation may, in many cases, be rendered unnecessary by thus pushing the point a little further into the soft bony substance. However skillfully the saw may be applied from beneath, in order to leave a pendant flap to cover the wound after the excised bone has been peeled out, more or less cicatricial tissue will be the result. So I think the cautery is a better means to this end, even than this best of all sawing operations, provided the bone is soft enough (and this often is the case) to be pierced by the white point, and even should the bone be too hard for puncture, the saw can be used to a better advantage after its thickened covering has been reduced, should it then be necessary.

So-called catarrhal neuralgias can often be permanently relieved by the deep needle puncture when the cautery cut or surface searing would fail. Any thickening of the superior turbinals and adjacent tissues surrounding the ethmoid, sphenoid and frontal sinus orifices, may so occlude any of them as to cause a damming of the normal secretions, with a dull heavy pain in the brow and between the eyes as the very natural sequence. The diagnostician should bear n mind the characteristic reflex pains in each of these sinuses: The pain from the ethmoidal is in the temples and between the eyes; from the sphenoidal it is referred to the occiput and to the deep nasal region; and from the frontal sinuses it is referred to the bridge of the nose and to the brow; but in all of these there is always a more or less dull heavy feeling between the orbits.

Again, the direct pressure of the swelling alone, in this narrowest part of the nasal cavity, on the sensitive nerve terminals will cause a reflex frontal and intraorbital pain in many nervous subjects. The partial, or complete, loss of smell is another frequent result of pressure in this olfactory region. Here the deep needle puncture can be safely used and with surprisingly good results, when to cut, or sear the surface with the cautery, or with caustics, would probably result in adhesions and in the permanent loss of olfaction. Such are the cases when not properly treated, that may finally require chiseling through the outer bony walls, leaving an ugly wound that can not be closed until the drainage through the natural channel is reestablished. Were it not better to have done the last first, by resorting to the proper means to that end in the beginning and thus avoid, at least, the probable necessity of a defacing operation? Some writers dwell upon the difficulties of the treatment of the upper sinus complications by the natural way. This method is too tedious in large clinics, and as the cosmetic consideration is secondary, the external operation is quite naturally resorted to, as the quickest and most spectacular means for immediate results. Hence the needle puncture in this region is not suggestive for clinical display.*

In preparing for the operation the seat of the application should first be cleansed with an antiseptic solution, then apply some one of the suprarenal extracts (yet I have sometimes felt that the blanching effect of the suprarenals may be carried too far, as the small bloodless tract of the needle or blade may afterwards serve as a channel of infection ; hence I prefer a little after-oozing, in which case the uncomfortable antiseptic plug may not always be necessary). After this the part is anesthetized with cocaine, beta-eucane, orthoform, or any other of the local anesthetics, and, as in mucous membranes, the process of osmosis is now reversed, at least exosmosis is checked, anesthesia is reached sooner and with a less quantity.

It may not be amiss here to say something of the fallacy of depending on sprays for the cure of real nasal and nasopharingeal troubles, when such dependence alone can only be placed in cases of the simplest acute inflammations of mucous membrane linings. Turbinal hypertrophies, hyperplasias, septal deviations and spurs, polyps, adenoids, etc., are local conditions in these cavities, and any spray directed to such localized lesions reaches also the other and more healthy parts, which can bear only the weaker spray solutions—too weak to make any decided effect on the diseased areas. It is true that a spray containing some local anesthetics, combined with a suprarenal extract, will give temporary

*An opportunity for original research is open to some one in the South especially, since it is asserted, but always vaguely, that the negro has none of the upper accessory sinuses, and that even air or pith cells are not always found in his frontal, ethmoid, or sphenoid bones(?). It is also tacitly conceded that the babe is born without cavities in those bones(?). So it would seem that nature's efforts at strength and symmetry through tubal economy is exerted after birth, and is reached only in delicately developed bony frames. On the other hand, could we not infer that nature sacrifices tubal lightness for solid strength to better resist external compact; i. e., that the lower species may the better meet blows from the front? At any rate we only find the upper sinus complications, with all their varied and far-reaching reflexes, in highly organized subjects, in whom these cavities are accessory to the nasal opening. Unfortunately, they are also dangerously near the meninges when infected, and with no natural exit through their tubes on account of the pressure in the region of the superior turbinates.

relief, and I am told that this is the daily practice of some, yet I can not imagine how they induce their patients to return daily, and for so long a time, unless it is that the patient soon acquires what might be called the coco-suprarenal-spray habit. Such sprays produce a short general exhilaration and a temporary respite from local discomfort or pain. But this method is like playing with fire, since at its cessation, or even during its use, recurrences are liable, and often with increased severity.

I am not prepared to make the statement from statistical evidence that complications of the nasal accessory sinuses—the antrum, the sphenoid, the frontal, the ethmoid, and even the mastoid through the middle ear—are more likely to occur in those who have been daily treated with sprays of cocaine and suprarenal extracts, yet I am impressed with the fact that I see proportionately more of these complications during the last few years, very many of which have the daily spray history. May this not be due to the patulous condition of the sinous openings produced by cocaine and adrenalin upon the normal, or relatively healthy, tissues that immediately surround these openings? Certainly septic matter could thus more easily reach these normally well-protected cavities to produce an infection in their less resisting mucous linings. I have made this seeming digression, not to condemn the spray, but on the contrary to suggest its correlation to the more potent means of reaching a better final result. Sprays are necessary to him who treats the upper respiratory tract—as necessary as are bandages and antiseptics to the general surgeon. Both are adjuncts, and neither can be relied upon alone only in the simpler cases.

Most local diseases incident to the upper respiratory tract are now amenable to permanent cure. Especially is this true in overgrowths—hypertrophies and hyperplasias—and in such cases there is probably no better instrument, means or method than the galvano-cautery. I mean, of course, minus its abuses and plus its proper uses. I have used the cautery some thousands of times; I could not now approximate the number from my incomplete records, but this I do know, that I have never once had an unsatisfactory result after properly using it, and when I have had the opportunity of repeating it as I wished I have always had good results. The operation can be done almost without pain, and so nearly is this true that, with tact, there is no difficulty in repeating it even in small children.

The longer I use it the more respect do I have for this cautery—as much for its possibilities of doing harm when misused as for the good it can do when properly used—and I feel now that I can more easily avoid its evils, as I am more confident of making it do what I would have it do. The galvano-cautery does not beg the question. It only asks that it be used in properly selected cases, and that it be rightly applied, then it will do all, and often more than is expected of it.

(Demonstrations were then made before the Atlanta Society of Medicine of the technique of application in the nose, in the throat, in the ear, in corneal and vocal-chord ulcers and in pterigium operations. Also the various shapes of the cautery points and blades, and the different degrees of heat used in each operation were demonstrated.)

*805-807 English-American Building.*

SOME BIBLIOGRAPHY.

Fraenkel, Vienna; Zuckerkandi, Berlin; Onodi, Austria; Luc, Paris; Sattler, Cincinnati; Jansen, Berlin; Hirschberg, Berlin; Myers, New York; Bosworth, New York; Solis-Cohen, Philadelphia; Mackenzie, Baltimore; Rokitansky, Vienna; Senne, Paris; Galezoskey, Paris; Bishop, Chicago; Landolt, Paris; Pritchett, London; Sorrotti, Barcelona; etc.

---

FOR a single intravenous infusion, as to combat the shock of hemorrhage, it is not essential that the solution contain any of the blood salts but the most abundant one—sodium chloride. For repeated infusions, however, as sometimes used in treating various toxemias, it is better to employ also the other salts, the solution being made of sodium chloride 0.9, potassium chloride 0.03, calcium choride 0.02, water 100.—*American Journal of Surgery.*

---

THE Nobel Prize committee is understood to have decided to award the prize for medicine to Prof. Robert Koch.

## Figures and Tables

**Figs. 1. and 2. f1:**
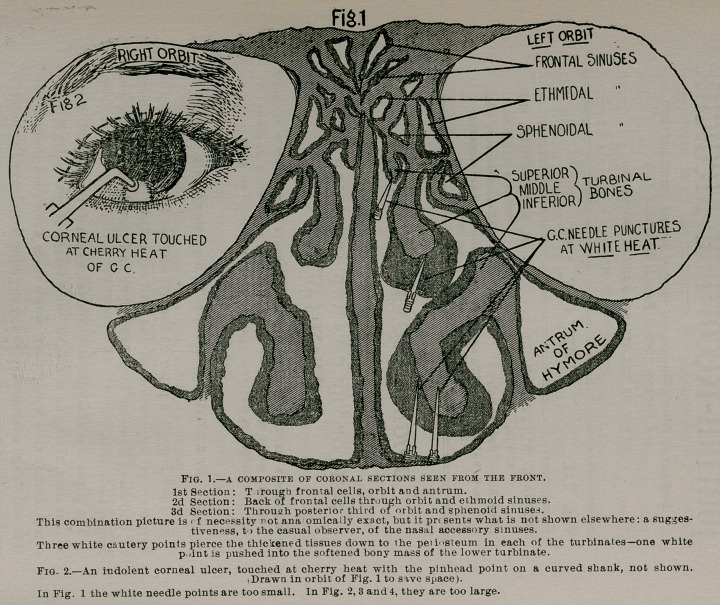


**Figs. 3. and 4. f2:**